# Oligonucleotide indexing of DNA barcodes: identification of tuna and other scombrid species in food products

**DOI:** 10.1186/1472-6750-10-60

**Published:** 2010-08-23

**Authors:** Sara Botti, Elisabetta Giuffra

**Affiliations:** 1 Parco Tecnologico Padano, CERSA - Centro Ricerche e Studi Agroalimentari, Via A. Einstein, 26900 Lodi, Italy

## Abstract

**Background:**

DNA barcodes are a global standard for species identification and have countless applications in the medical, forensic and alimentary fields, but few barcoding methods work efficiently in samples in which DNA is degraded, e.g. foods and archival specimens. This limits the choice of target regions harbouring a sufficient number of diagnostic polymorphisms. The method described here uses existing PCR and sequencing methodologies to detect mitochondrial DNA polymorphisms in complex matrices such as foods. The reported application allowed the discrimination among 17 fish species of the Scombridae family with high commercial interest such as mackerels, bonitos and tunas which are often present in processed seafood. The approach can be easily upgraded with the release of new genetic diversity information to increase the range of detected species.

**Results:**

Cocktail of primers are designed for PCR using publicly available sequences of the target sequence. They are composed of a fixed 5' region and of variable 3' cocktail portions that allow amplification of any member of a group of species of interest. The population of short amplicons is directly sequenced and indexed using primers containing a longer 5' region and the non polymorphic portion of the cocktail portion. A 226 bp region of *CytB *was selected as target after collection and screening of 148 online sequences; 85 SNPs were found, of which 75 were present in at least two sequences. Primers were also designed for two shorter sub-fragments that could be amplified from highly degraded samples. The test was used on 103 samples of seafood (canned tuna and scomber, tuna salad, tuna sauce) and could successfully detect the presence of different or additional species that were not identified on the labelling of canned tuna, tuna salad and sauce samples.

**Conclusions:**

The described method is largely independent of the degree of degradation of DNA source and can thus be applied to processed seafood. Moreover, the method is highly flexible: publicly available sequence information on mitochondrial genomes are rapidly increasing for most species, facilitating the choice of target sequences and the improvement of resolution of the test. This is particularly important for discrimination of marine and aquaculture species for which genome information is still limited.

## Background

In DNA barcoding, a polymorphic DNA sequence from a standardized and agreed-upon position in the mitochondrial genome is used as a molecular diagnostic for species-level identification. DNA barcodes are being increasingly used as a global standard for species identification and biodiversity studies, and have many potential applications in the medical, forensic and alimentary fields http://www.barcoding.si.edu.

One of the purposes of DNA barcodes is to provide unambiguous references to quickly identify undesirable animal or plant material in processed foods and to detect commercial products derived from regulated species [1]. However, few methods for the detection and discrimination of animal species-specific ingredients work efficiently starting from foodstuff. The conservation of meat is often based on prolonged cooking, processing or autoclaving, which cause DNA degradation and limits the choice of target regions harbouring useful diagnostic polymorphisms. For this reason, in addition to the standard barcode in use for the identification of many animal species (a 648 bp region of the mitochondrial *cytochrome c oxidase 1 *gene, *COI *[2]), the use of universal mini-barcodes has been proposed for use with archived and environmentally derived specimens (e.g. faeces) in biodiversity studies [3].

The family Scombridae contains 15 genera and about 51 species of epipelagic and generally migratory marine fish. It includes species with a high commercial interest such as mackerels, bonitos, and tunas, of which nearly 9 million tons were caught world-wide in 2007 [4,5]. The geographic distribution of the individual species differs, as do their commercial value, and related ecological importance (e.g. the Atlantic bluefin tuna is in danger of extinction). Many of these fish are present as the main or secondary ingredient in various foods which are prone to frauds. Population genetic and biodiversity studies of these species, mostly based on polymorphisms occurring in repetitive genomic regions (mtDNA, ribosomal genes), have provided the reference knowledge to develop molecular tests for species identification [6,7]. However, only few of the recently reported methods work efficiently for species traceability in foods, and in general these tests discriminate among few species.

The method described here uses existing and easily automated methodologies (PCR, sequencing) to detect any mitochondrial DNA polymorphisms in complex matrices such as food. The application of the method demonstrates the discrimination of 17 species of the Scombridae family, which are often present in processed seafood. The test could be easily upgraded with the release of online genetic diversity information to improve the power and range of species detected.

## Results

### Method of oligonucleotide indexing

**Figure 1:**
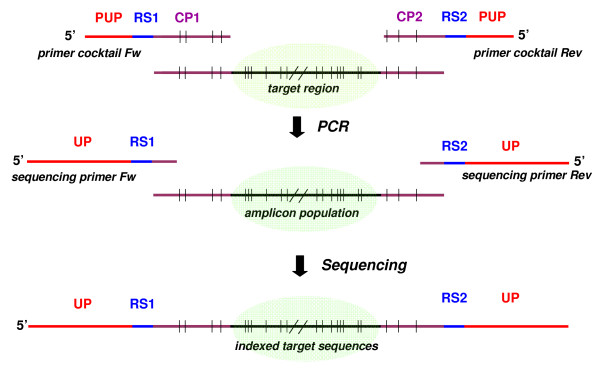
**Scheme of the method of oligonucleotide indexing**. PUP: Partial Universal Primer; RS: Read Start sequence; CP: Cocktail Portion; UP: Universal Primer. Sequence polymorphisms are represented by vertical bars; the ones localized in the cocktail portion are used to design the primer cocktail composition and the sequencing primers (Table 2). The PCR generates an amplicon population; each individual amplicon harbours species-specific polymorphisms in the target region (shadowed). Individual amplicons are sequenced using the sequencing primers. Sequences are aligned using the read start reference and analyzed to determine the species content.

Figure 1 shows the principle of the method. A cocktail of oligonucleotides for PCR is constructed using publicly available sequences of the target mitochondrial sequence and surrounding regions. The structure of the primers composing the cocktail includes (from 5' to 3'): a portion of a universal sequencing primer, e.g. M13 (partial universal primer, PUP), a 'read start' portion (RS), and a portion complementary to the region flanking the target sequence (cocktail portion, CP). The PUP region facilitates the subsequent reading of the full target sequence from a single strand by elongating the amplified PCR fragment. The RS is a strand- specific, arbitrary fragment of 5-6 nucleotides included to ease sequence interpretation for each strand, i.e. once the PCR product is sequenced, the raw sequences can be trimmed, aligned and analysed from the same RS. The composition of the 3' regions of the forward and reverse cocktail portions is determined on the basis of the known polymorphisms flanking the target region in order to amplify any member of the group of species of interest, e.g. species which could be possibly present in a given food matrix. The amplicon population resulting from the PCR products obtained is sequenced from either strand using primers containing a longer universal primer region (UP) at the 5', the RS, and the non polymorphic portion of the CP region at the 3' (Figure 1). Raw sequences are trimmed, aligned and analysed by standard sequence analysis tools [8,9].

### Demonstration application: discrimination of tuna and scombrid species in food products

The method was validated on a group of species which are extensively used as main or secondary ingredients in foodstuff, namely the Scombridae fish family (Table 1). The mitochondrial *CytB *gene was chosen as the target because of the abundant reference sequence data for most species of this family. Alignment of 148 online sequences allowed to target a fragment of 226 bp mapping in position 14652 - 14877 of the mitochondrial genome of *Thunnus thynnus *[NCBI: NC_004901]. A total of 85 SNPs were identified, of which 75 were present in at least two sequences (Additional files 1, 2, and 3). The net average numbers of base differences in pairwise sequence comparison reflected the different extent of genetic divergence within and between taxonomical groups (Additional file 4). The highest value (37.9 ± 4.5) differentiated *T. tonggol *vs. *S. colias*. Within the eight tuna species, values ranged between 1.1 ± 1.0 for the pair of closely related species *T. thynnus *vs. *T. maccoyii *and 10.7 ± 2.7 (*T. tonggol *vs. *T. orientalis*). In mackerels, these values ranged between 1.7 ± 1.1 for *S. japonicus *vs. *S. australasicus *and 28.7 ± 3.9 (*S. scombrus *vs. *S. colias*). A value of 12.0 ± 3.0 differentiated the two *Auxis *species *(A. thazard thazard *vs. *A. rochei rochei*).

**Table 1:** List of fish species (Scombridae) which can be discriminated in seafood by the present method.

*Scientific Name*	*Common name*	*Acronym*
*Thunnus thynnus*	Atlantic bluefin tuna	TTHY
*Thunnus albacares*	Yellowfin tuna	TALB
*Thunnus atlanticus*	Blackfin tuna	TATL
*Thunnus maccoyii*	Southern bluefin tuna	TMAC
*Thunnus orientalis*	Pacific bluefin tuna	TORI
*Thunnus obesus*	Bigeye tuna	TOBE
*Thunnus alalunga*	Albacore	TALA
*Thunnus tonggol*	Longtail tuna	TTON
*Katsuwonus pelamis*	Skipjack tuna	KPEL
*Euthynnus alletteratus*	Little tunny	EALL
*Auxis thazard thazard*	Frigate tuna	ATHA
*Auxis rochei rochei*	Bullet tuna	AROC
*Sarda sarda*	Atlantic bonito	SSAR
*Scomber scombrus*	Atlantic mackerel	SSCO
*Scomber japonicus*	Chub mackerel	SJAP
*Scomber colias*	Atlantic chub mackerel	SCOL
*Scomber australasicus*	Blue mackerel	SAUS

For most food samples the quality of the extracted DNA was sufficient to amplify this fragment size (226 bp). In order to achieve amplification of much degraded samples, additional primer cocktails were designed to amplify two smaller fragments (A: 109 bp containing 45 SNPs; B: 95 bp containing 36 SNPs) mapping respectively on intervals 14652 - 14760 and 14783 - 14877 of *Thunnus thynnus *Reference Sequence [NCBI: NC_004901]. Primer cocktails and sequencing primers were designed on the basis of the scheme in Figure 1 and are provided in Table 2. For the primer cocktails, the primer regions complementary to the mitochondrial target were designed to include all the SNPs described for this group of species. For closely related species, a diagnostic value was given only to the sites that were polymorphic between the species that did not present intraspecific variation [10].

**Table 2:** Primer cocktail (F, forward and R, reverse) and sequencing primers to detect polymorphisms occurring in fragments AB (226 bp), A (109 bp) and B (95 bp) of mitochondrial *cytochrome b *in 17 fish species of the Scombridae fish family.

*Primer name*	Primer sequence 5'-3'
***PCR primer cocktails***	***FRAGMENT AB***
USBotA-CytbsF	*GCCAGT***GATTAC**AACGGGGCCTCTTTCTTCTT
USBotB-CytbsF	*GCCAGT***GATTAC**AACGGCGCYTCCTTCTTCTT
USBotCbis-CytbsF	*GCCAGT***GATTAC**AACGGTGCTTCYTTCTTCTT
USBotDbis-CytbsF	*GCCAGT***GATTAC**AACGGGGCCTCCTTCTTCTT
USS1-UniT-R	*TGACC***CGATC**GTRGCATTGTCTACTGARAAGCC
USS1-UniK-R	*TGACC***CGATC**GTGGCGTTGTCTACTGAAAAGCC
USS1-UniR-R	*TGACC***CGATC**GTGGCGTTGTCTACTGAGAAGCC
USS1-UniSA-R	*TGACC***CGATC**GTRGCATTGTCTACAGAGAAGCC
***Sequencing primers***	
USbot2-F	*GTTGTAAAACGACGGCCAGT***GATTAC**AACGG
USS2-UniT-R	*CACAGGAAACAGCTATGACC***CGATC**GTRGC
	
***PCR primer cocktails***	***FRAGMENT A***
USBotA-CytbsF	*GCCAGT***GATTAC**AACGGGGCCTCTTTCTTCTT
USBotB-CytbsF	*GCCAGT***GATTAC**AACGGCGCYTCCTTCTTCTT
USBotCbis-CytbsF	*GCCAGT***GATTAC**AACGGTGCTTCYTTCTTCTT
USBotDbis-CytbsF	*GCCAGT***GATTAC**AACGGGGCCTCCTTCTTCTT
USAP-UniT-R	*ATGACC***GACCA**AGGGAAGRACGTAGCCRACGAA
USAP-UniK-R	*ATGACC***GACCA**AGGGAAGTACGTARCCGACGAA
USAP-UniR-R	*ATGACC***GACCA**ATGGRAGTACGTAGCCGACGAA
USAP-UniE-R	*ATGACC***GACCA**AAGGGAGTACGTAACCGACGAA
USAP-UniSA-R	*ATGACC***GACCA**AGGGAAGRACGTARCCGACGAA
***Sequencing primers***	
USbot2-F	*GTTGTAAAACGACGGCCAGT***GATTAC**AACGG
USAP2-R	*CACAGGAAACAGCTATGACC***GACCA**ANGG
	
***PCR primer cocktails***	***FRAGMENT B***
USS1-UniT-F	*GCCAGT***GATTAC**TTCGTYGGCTACGTYCTTCCCT
USS1-UniK-F	*GCCAGT***GATTAC**TTCGTCGGYTACGTACTTCCCT
USS1-UniR-F	*GCCAGT***GATTAC**TTCGTCGGCTACGTACTYCCAT
USS1-UniE-F	*GCCAGT***GATTAC**TTCGTCGGTTACGTACTCCCTT
USS1-UniSA-F	*GCCAGT***GATTAC**TTCGTCGGYTACGTYCTTCCCT
USS1-UniT-R	*TGACC***CGATC**GTRGCATTGTCTACTGARAAGCC
USS1-UniK-R	*TGACC***CGATC**GTGGCGTTGTCTACTGAAAAGCC
USS1-UniR-R	*TGACC***CGATC**GTGGCGTTGTCTACTGAGAAGCC
USS1-UniSA-R	*TGACC***CGATC**GTRGCATTGTCTACAGAGAAGCC
***Sequencing primers***	
USS2-UniT-F	*GTTGTAAAACGACGGCCAGT***GATTAC**TTCGT
USS2-UniT-R	*CACAGGAAACAGCTATGACC***CGATC**GTRGC

The amplicon size is respectively of 291 bp (fragment AB), 175 bp (fragment A) and 163 bp (fragment B). Italic: 5' Partial Universal Primer (PUP) and 5' Universal Primer (UP); bold: Read Start signal (RS1 and RS2); normal text: 3' cocktail portion (CP).

The test was applied to verify the species content of 203 samples of fish-containing foods (canned tuna and scomber, tuna salad, tuna sauce) provided by industrial retailers (Table 3). Seven out of ten batches of canned tuna were found to be consistent with the single species declared on product label (*T. albacares*). A single batch composed of 100 individual samples of canned tuna was found to contain 2 samples of *T. albacares*, 72 of *T. obesus*, 5 of *K. pelamis*, and 19 and 2 samples representing a mix of *T. obesus/K. pelamis*. Two *Thunnus *variants found in two batches of 10 samples could not be matched to any known entry in the online database and represent new polymorphisms of *Thunnus *spp. Only fragments A and B could be successfully amplified in tuna salad (2 samples) and tuna sauce (8 samples), this was probably due to extensive DNA degradation of these products. In both cases this was sufficient to detect differences in species content from what declared on the product labels: for tuna salad samples, the test revealed in both samples the presence of *Katsuwonus pelamis *instead of *T. albacares*, while in all the eight samples of tuna sauce the test revealed a mix of multiple species not compatible with the declared presence of the only *T. albacares *variants (Table 3).

**Table 3:** Application of the described methods: species discrimination of tuna and scomber species in food product.

*Sample*	*Species Declared*	*N° of samples/batch*	*Species identified (fragment AB)*
Canned tuna		10	*10 T. albacares*
		
		10	*9 T. albacares*
			*1 T. thynnus*
		
		10	*10 T. albacares*
		
	*T. albacares*	100	*2 T. albacares*
			*72 T. obesus*
			*5 K. pelamis*
			*19 mix T. obesus/K. pelamis*
			*2 mix T. albacares/T. obesus*
		
		10	*10 T. albacares*
		
		10	*10 T. albacares*
		
		10	*10 T. albacares*
		
		10	*9 T. albacares*
			*1 new variant (Thunnus spp.)*
		
		10	*9 T. albacares*
			*1 new variant (Thunnus spp.)*
		
		8	*8 T. albacares*
		
		1	*1 T. obesus*
		
		2	*2 T. albacares*

Tuna salad	*T. albacares*	2	(*) 2 K. pelamis

Tuna sauce (20% tuna)	*T. albacares*	8	(*) Mix of species *(different genera)*

Canned scomber	*S. japonicus*	2	*2 S. colias*

(*) Only fragment B

**A total of 203 samples of processed food were delivered in 15 batches by industrial retailers for the control of species content and analyzed for polymorphism in fragment AB of *CytB***.

## Discussion

The method described here is based on cocktails of forward and reverse oligonucleotides for PCR which are designed to identify and index the genetic diversity known to occur at a target region in any species of interest. The use of a primer cocktail expands considerably the choice of the polymorphic target sequence because the target does not need to be flanked by fixed regions, which is the required condition to design conventional primers. This allows choosing target sequences which are sufficiently short to make the test efficient for transformed products in which DNA is degraded. The fixed 5' regions of the primers for PCR is sufficiently long to allow sequencing of small fragments (< 100 bp) without the need of cloning. Using 'read start' sequences as indexing markers can be a useful for fast alignment and identification of the target of different species in high throughput applications (Figure 1).

The method relies on the existing information in databases of genomic diversity. With the increasing use of massively parallel sequencing and falling costs the publicly available sequence information is growing rapidly. The information on the SNPs occurring in the variable portion of the primer cocktail can be easily upgraded to include additional variants of the same species and of new ones. Moreover, informatics tools become increasingly available and facilitate the automation and indexing of DNA sequence analyses deposited in online or custom databases without the need of sophisticated informatics know how [11]. Remarkably, in the case of the *COI *gene it was computationally predicted that 'universal' fragments of 100 bp and 250 bp should have respectively 90% and 95% probabilities to discriminate most animal species [3].

In the case application illustrated in this paper a 226 bp region of *CytB *was chosen on the basis of online sequence information (Table 1, Table 2, Additional file 1, Additional file 2, Additional file 3) with the objective of designing an assay to identify tuna and scomber species widely used for seafood by the identification of mitochondrial haplotypes. This fragment size proved to be short enough for efficient PCR amplification of degraded DNA samples obtained from canned tuna and scomber samples. Depending on the online information on the position of putative diagnostic SNPs in the 226 bp AB fragment, either fragment B or A can allow discriminating most member of this group of Scombridae (Additional file 4), with few exceptions for the most closely *Thunnus *species (see below). When only the B fragment was efficiently amplified and sequenced, the polymorphism of this small fragment (95 bp) was sufficient to detect a gross mislabelling of species content in the mostly degraded samples such as tuna salad and tuna sauce (Table 3).

The method constitutes a significant improvement of several previously described tests for the control and traceability of tuna-containing food [12-15] which in general are designed for longer target sequences, and/or can only discriminate small groups of taxonomically closely related species (e.g. *Thunnus *spp.). DNA extractions, PCR reactions and sequencing are performed by standard protocols, and are thus suited for large scale analyses which will contribute to enrich the database of Scombrid species with new markers of intra- and inter- specific variability.

Some technical and biological caveats in our methods need to be considered. From the technical point of view, to choose the best target fragment on the basis of described polymorphisms and to carry out the quality control of the online sequences are time consuming tasks. In our case, the *CytB *sequences which passed the quality control were found to be poorly represented in some of the target Scombridae species, thus decreasing the power to define diagnostic SNPs (Additional file 4). For example, a single diagnostic SNP could differentiate *T. thynnus *vs. *T. maccoyi *and *T. orientalis *vs. *T. alalunga*, but the online sequences representing *T. maccoyi *and *T. orientalis *were only four and three, respectively. From the biological point of view, some species are often too closely related to be discriminated using either short mitochondrial or nuclear markers, and in addition the introgression of mitochondrial genomes between close fish species is a well known phenomenon, e.g. among *Scomber colias, S. japonicus *and *S. australasicus *[16] and among species of *Thunnus *(e.g. between *T. thynnus *and *T. orientalis*; [17]). It has been recently shown that longer mtDNA target regions or the combination of mitochondrial and nuclear markers allow unambiguous discrimination between the eight species of the *Thunnus *complex [7,18]. However, most tests for validating foods need to be, at least for large scale product screenings, economically sustainable. Therefore a two step strategy may be considered, i.e. to use this rapid screening protocol to identify potential mislabelling and frauds followed by an additional focussed test of only the mislabelled samples. The latter might include the analysis of nuclear regions to exclude mitochondrial introgression of the closely related species, or cloning and sequencing of the mitochondrial fragments to discriminate multiple species contents and infer their relative amounts in the most problematic samples.

## Conclusions

The approach described here facilitates a highly flexible diagnostic procedure which is largely independent of the fragmentation of input DNA source. Publicly available sequence information on mitochondrial genomes are rapidly increasing for most species, facilitating the choice of the best target sequence and the improvement of the resolution of the test. These features are particularly important for the discrimination of species which are increasingly employed as food ingredients as it is the case for many marine and aquaculture species for which genome information is still limited.

## Methods

### Sample collection and DNA extraction

Samples of foods (canned fish either in water or oil, fish-containing sauces, tuna salads, tuna sauces) were purchased in commercial markets or provided by retailers for fraud testing (Table 2). Reference fish samples for multiplex PCR validation were obtained from the fresh fish market and included one specimen each of the following species: *Thunnus thynnus*, *Auxis rochei*, *Euthynnus alletteratus*, *Sarda sarda*, *Thunnus albacares*, and *Scomber scombrus*. Muscle samples were mashed and autoclaved for 30 minutes to cause extensive DNA degradation and mixed in different proportions prior DNA extraction. Samples of canned fish were placed on filter paper in order to remove the excess of oil or water.

DNA extractions were carried out using the General Rapid Easy Extraction System (GREES) DNA Kit (InCura Srl, Italy) following manufacturer's recommendations.

### Primer design, PCR conditions and sequencing

The available *CytB *sequences of commercial *Scombridae *species were downloaded from the NCBI http://www.ncbi.nlm.nih.gov "nucleotide" database and aligned by Clustal_X [8]. The estimates of net evolutionary divergence between the 17 target species was performed on a finals set of 148 sequences using MEGA [9]. Standard errors were obtained by a bootstrap procedure (1000 replicates). All positions containing alignment gaps and missing data were eliminated only in pairwise sequence comparisons (Pairwise deletion option).

The cocktail of forward and reverse primers used for PCR amplification and sequencing were constructed following the scheme in Figure 1. Each forward and reverse primer cocktail was composed of 4 or 5 individual primers, depending on the target fragment of PCR (Table 2). From 5' to 3', each primer for PCR was composed of the PUP (6 nucleotides at 3' of the universal primer M13), the RS (5-6 nucleotides) and the CP sequence (between 20 and 23 nucleotides). The sequencing primers were composed (from 5' to 3') of 20 nucleotide of M13, of the full RS, and of the first 5 nucleotides of the polymorphic target (in this case, *CytB*).

The PCR conditions to amplify the target regions of *CytB *were the same for the three fragments (AB, A and B). The reaction mixture (total of 20 μl) included 1.5 μl of DNA (20 ng/μl) as template, 375 nM of each individual primer (forward and reverse), 0.2 mM dNTPs, and 0.5 U of HotStartTaq DNA Polymerase (Qiagen). PCR reactions were prepared in 96 optical well plates using a TECAN FREEDOM EVO-150 liquid handling workstation (Tecan Trading AG, Switzerland) and run in a Peltier Thermal Cycler PTC-200 (MJ-Research). The thermal PCR profile was 95°C for 15 min, followed by 35 cycles of: 95°C for 45 s, 60°C for 1 min and 72°C for 45 s, followed by final elongation step at 72°C for 10 min.

Sequencing was carried out with the ABI PRISM BigDye3.1 Terminator Cycle Sequencing Kit (Applied Biosystems) following the manufacturer's recommendations. When DNAs from two different species are mixed in different proportions, and the less abundant species is represented at 5% or more, both sequence profiles are clearly detected (data not shown).

## Authors' contributions

SB conceived and designed the barcoding method, analyzed the results, carried out the experimental work and contributed to draft the manuscript. EG supervised the design of the study and drafted this manuscript. Both authors read and approved the final manuscript.

## Supplementary Material

Additional file 1 **Alignment of the 85 SNPs found in fragment AB of *CytB *of the fish family Scombridae.** The first 49 SNPs are found in fragment A, while SNPs 50 - 85 are present in fragment B. Acronym of species as in Table 1 of manuscript. Forty-two tunas representing eight species of the *Thunnus *species complex Reference sequences: *Thunnus thynnus *[NCBI: NC_004901; portion 14652-14877 bp]. Click here for file

Additional file 2 **Alignment of the 85 SNPs found in fragment AB of *CytB *of the fish family Scombridae.** The first 49 SNPs are found in fragment A, while SNPs 50 - 85 are present in fragment B. Acronym of species as in Table 1 of manuscript. Twenty-eight mackerels (*Scomber *spp.). Reference sequence: *Scomber japonicus *[NCBI: AB018996] Click here for file

Additional file 3 **Alignment of the 85 SNPs found in fragment AB of *CytB *of the fish family Scombridae.** The first 49 SNPs are found in fragment A, while SNPs 50 - 85 are present in fragment B. Acronym of species as in Table 1 of manuscript. Seventy-eight fish representing frigate tuna (*Auxis thazard thazard*), bullet tuna (*Auxis rochei rochei*), Atlantic bonito (*Sarda sarda*), little tunny (*Euthynnus alletteratus)*, skipjack tuna (*Katsuwonus pelamis*). Reference sequence: *Auxis thazard thazard *[NCBI: DQ080314]. Click here for file

Additional file 4 **Estimates of Net Evolutionary Divergence between Groups of Sequences**. The number of base differences per sequence from estimation of net average between groups of sequences is shown. All results are based on the pairwise analysis of 151 sequences containing 85 variable positions. Standard error estimates are shown in the second column and were obtained by a bootstrap procedure (1000 replicates). Click here for file
