# Supplementary material for: Oligonucleotide indexing of DNA barcodes: identification of tuna and other scombrid species in food products
Source: BMC Biotechnol. 2010 Aug 23;10:60. doi: 10.1186/1472-6750-10-60 (PMC2936417; doi:10.1186/1472-6750-10-60)
Supplement: Additional file 2 — Alignment of the 85 SNPs found in fragment AB of CytB of the fish family Scombridae. The first 49 SNPs are found in fragment A, while SNPs 50 - 85 are present in fragment B. Acronym of species as in Table 1 of manuscript. Twenty-eight mackerels (Scomber spp.). Reference sequence: Scomber japonicus [NCBI: AB018996] [file 1472-6750-10-60-S2.PDF]

```
                10        20        30        40        50        60        70        80
        ....|....|....|....|....|....|....|....|....|....|....|....|....|....|....|....|....|
SJAP1   CTCATTCTCCCGCTCCCCTTCATGACATTGGTTTCCCCAAACACCCCTCACCAGCCTCAGCCCTATACTGACACCTTCCTGACAC
SJAP2   .................................................................C...................
SJAP3   ---------------------................................................................
SJAP4   --------------------.................................................A...............
SJAP5   ............T..T....T...G.........................T...........T.GC..C.....T..A.......
SCOL1   ...........AT..T.............A....................T........A..T.GC..C........A.......
SCOL2   ............T..T..................................T........A..T.GC..C.....T..A.......
SCOL3   ............T..T.............A....................T........A..T.GC..C.....T..A.......
SCOL4   ............T..T.............A....T...............T........A..T..C..CA.......A.......
SCOL5   ............T..T.............A....................T........A..T.GC..C........A.......
SCOL6   ............T..T.............A.............................A..T..C..C........A.......
SCOL7   ...........AT..T.............A....................T........A..T.GC..C.....T..A.......
SCOL8   ............T..T.............C....................T........A..T.GC..C.....T..A.......
SCOL9   ............T..T.............C....................T...........T.GC..C........A.......
SCOL10  ............T..TT............A....................T........A..T.....C........A.......
SAUS1   ....C..........T.......A.....A.............G...--------------------------------------
SAUS2   ...............T.............A...................................C...........A.....G.
SAUS3   ...............T.............A...................................C...........A.......
SAUS4   ...............T.............A...................................C..C........A.....G.
SAUS6   ----------------------------.A.......................A...........C........A..A.......
SAUS7   --------------------.........A...................................C...........A.....G.
SAUS8   ----------------------------.A....................T........A..T..C...........A.......
SSCO1   ...CCC.....A.G..A...TG.AG.GC.A..CC.....GGTCT......A...........TCTC..CA.T..ACAA..A.T.G
SSCO2   ...CCC.....A.G..A...TG.AG.GC.A..CC.....GGTCT......A...........TCTC..CA.T..ACAA..A.T.G
SSCO3   ...CCC.....A.G..A...TG.AG.GC.A..CC.....GGTCT......A...........TCTC..CA.T..ACAA..A.T.G
SSCO4   ...CCC.....A.G..A...TG.AG.GC.A..CC.....GGTCT......A...........TCTC..CA.T..ACAA..AGT.G
SSCO5   ----------------------.AG.GC.A..CC.....GGTCT......A...........TCTC..CA.T..ACAA..A.T.G
SSCO6   ...C.......A.G..A...TG.AG.GC.A..CC.....GGTCT......A........A..TCTC..CA.T..ACAA..A.T.G
```
