# Supplementary material for: Oligonucleotide indexing of DNA barcodes: identification of tuna and other scombrid species in food products
Source: BMC Biotechnol. 2010 Aug 23;10:60. doi: 10.1186/1472-6750-10-60 (PMC2936417; doi:10.1186/1472-6750-10-60)
Supplement: Additional file 3 — Alignment of the 85 SNPs found in fragment AB of CytB of the fish family Scombridae. The first 49 SNPs are found in fragment A, while SNPs 50 - 85 are present in fragment B. Acronym of species as in Table 1 of manuscript. Seventy-eight fish representing frigate tuna (Auxis thazard thazard), bullet tuna (Auxis rochei rochei), Atlantic bonito (Sarda sarda), little tunny (Euthynnus alletteratus), skipjack tuna (Katsuwonus pelamis). Reference sequence: Auxis thazard thazard [NCBI: DQ080314]. [file 1472-6750-10-60-S3.PDF]

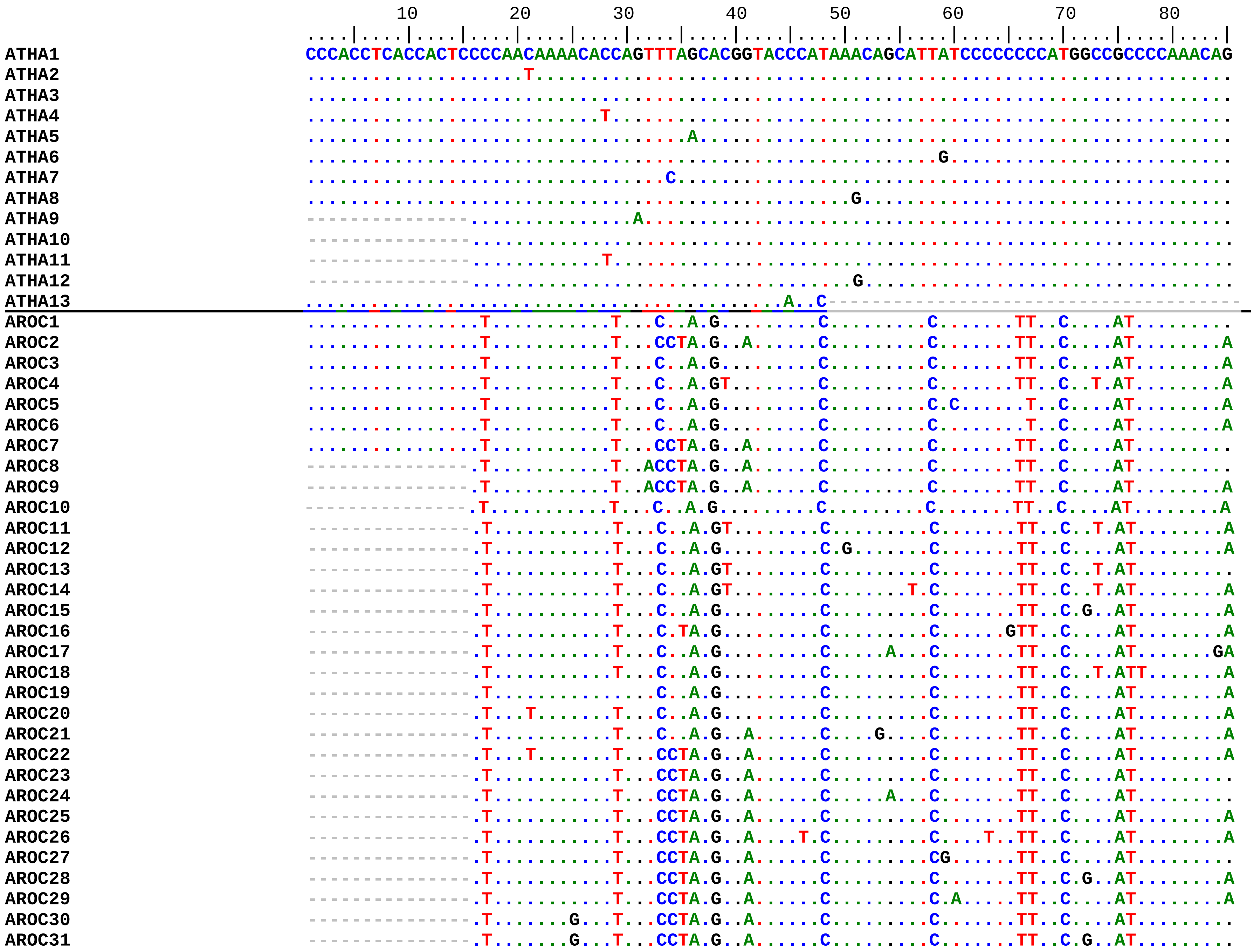

10 20 30 40 50 60 70 80
ATHA1 CCCACCTCACCACTCCCCAACAAAACACCAGTTTAGCACGGTACCCATAAACAGCATTATCCCCCCCCATGGCCGCCCCAAACAG
ATHA2
ATHA3
ATHA4
ATHA5
ATHA6
ATHA7
ATHA8
ATHA9
ATHA10
ATHA11
ATHA12
ATHA13
AROC1
AROC2
AROC3
AROC4
AROC5
AROC6
AROC7
AROC8
AROC9
AROC10
AROC11
AROC12
AROC13
AROC14
AROC15
AROC16
AROC17
AROC18
AROC19
AROC20
AROC21
AROC22
AROC23
AROC24
AROC25
AROC26
AROC27
AROC28
AROC29
AROC30
AROC31

```
AROC32   ---------------.T...........T...CCTA.G..A......C.........CG......TT..C....AT........A
AROC33   ................T...........T...C..A.G.........C.........C.......TT..C..T.ATT.......A
AROC34   ................T...........T...C..A.GT........C.........C.......TT..C..T.AT.........
AROC35   ............................T...C..A.G.........C.........C.......TT..C.G..AT........A
AROC36   ................T...........T...C..A.G.........C.........C.......TT..C.G..AT........A
SSAR1    .......TCTTT...............T....CC.TT.A..CT...T.C...G..C.C.GT.......CC..A.AAT..C.....
SSAR2    ..T....TC.TC........T......T....C..TT.A..CT.T.T.C...G..C.C.AT.......CC....AAT..C.....
SSAR3    ..T....TC.TC...............T....C..TT.A..CT.T.T.C...G..C.C.AT.......CC....AAT..C.....
SSAR4    .......TCTTT...............TA...CC.TT.A..CT...T.C...G..C.C.GT.......CC..A.AAT..C.....
SSAR5    .......TCTTT...............TA...CC.TT.A..CT...C.C...G..C.C.GT.......CC..A.AAT..C.....
EALL1    .T..T......CT...T.T........TT...A..C..A.ACC.T..CT.........C.....TT.TT..AA..AT..TC..T..
EALL2    ---------------.T.T........TT...A..C..A.ACC.T..CT.........C.....TT.TT..AA..AT..TC..T..
EALL3    .T..T......CT...T.T........TT...A..C..A.ACC....CT......C.C..T..TT...TCAA..AA..TC.....
KPEL1    ....T...CTTCTC.TA.C.........T....ATC..A...T..------------------------------------------
KPEL2    T...T...C.TCTC.T..C.........T...CATC..A...T.....C........C.AT..T..T..CAAA.AAT..C.....
KPEL3    ....T...CTTCTC.T..C.........T...CATC..A...T.....C.G......C.AT..T..T..CAAA.AAT..C.....
KPEL4    ....T...CTTCTC.T..C.........T...CATC..A...T.....C........C.AT..T..T..CAAA.AAT..C....A
KPEL5    ....T...CTTCTC.T..C.........T...CATC..A...T.....C........C.AT..T..T..CAAA.AAT..C.....
KPEL6    ....T...CTTCTC.T..C.........T...CATC..A.A.T.....C.G......C.AT..T..T..CAAA.AAT..C....A
KPEL7    ....T...CTTCTC.T..C.........T...CATC..A...T.....C.G......C.AT..T..T..CAAA.AAT..C....A
KPEL8    ....T...CTTCTC.T..C......T..T...CATC..A...T.....C.G......C.AT..T..T..CAAA.AAT..C....A
KPEL9    ....T...CTTCTC.T..C.........T...CATC..A...T.....C.G......C.AT..T.....CAAA.AAT..C....A
KPEL10   ....T...C.TCTC.T..C.........T...CATC..A.A.T.....C.G......C.AT..T..T..CA.A.AAT..C....A
KPEL11   ....T...CTTCTC.T..C.........T...CATC..A...T.T...C........C.AT..T..T..CAAA.AAT..C.....
KPEL12   ....T...CTTCT..T..C.........T...CATC..A...T.....C.G......C.AT..T..T..CAAA.AAT..C....A
KPEL13   ....T...CTTCTC.T..C.........T...CATC..A...T.T...C........C.AT..T..T..CAAA.AAT..C....A
KPEL14   ....T...CTTCTC.T..C.........T...CATC..A...T.....C.G......C.AT..T..T..AAAA.AAT..C....A
KPEL15   ....T...CTTCTC.T..C.........A...CATC..A...T.....C.G......C.AT..T..T..CAAA.AAT..C....A
KPEL16   ---------------T..C.........T...CATC..A...T.....C.G......C.AT..T..T..CAAA.AAT..C.....
KPEL17   ---------------T..C.........T...CATC..A...T.....C.G......C.AT..T.TT..CAAA.AAT..C....A
KPEL18   ---------------T..C.........T...CATC..A...T.T...C........C.AT..T..T..CAAA.AAT..C....A
KPEL19   ....T...C.TCTC.T..C.........T...CATC..A...T.T...C........C.AT..T..T..CAAA.AAT..C....A
KPEL20   ....T...CTTCTC.T..C.........T....ATC..A...T.....C.G......C.AT..T..T..CAAA.AAT..C....A
KPEL21   ....T...CTTCTC.T..C.........T...CATC..A...T.....C.G......CGAT..T..T..CAAA.AAT..C....A
```
