# Supplementary material for: Oligonucleotide indexing of DNA barcodes: identification of tuna and other scombrid species in food products
Source: BMC Biotechnol. 2010 Aug 23;10:60. doi: 10.1186/1472-6750-10-60 (PMC2936417; doi:10.1186/1472-6750-10-60)
Supplement: Additional file 4 — Estimates of Net Evolutionary Divergence between Groups of Sequences. The number of base differences per sequence from estimation of net average between groups of sequences is shown. All results are based on the pairwise analysis of 151 sequences containing 85 variable positions. Standard error estimates are shown in the second column and were obtained by a bootstrap procedure (1000 replicates). [file 1472-6750-10-60-S4.PDF]

	TTHY	TALB	TATL	TMAC	TORI	TALA	TTON	TOBE	KPEL	EALL	ATHA	AROC	SSAR	SSCO	SJAP	SCOL	SAUS
**TTHY**	-	-	-	-	-	-	-		-	-	-	-	-	-	-	-	-
**TALB**	3.3±1.5	-	-	-	-	-	-	-	-	-	-	-	-	-	-	-	-
**TATL**	1.5±1.1	1.1±1.2	-	-	-	-	-	-	-	-	-	-	-	-	-	-	-
**TMAC**	1.1±1.0	4.3±1.9	3.0±1.4	-	-	-	-	-	-	-	-	-	-	-	-	-	-
**TORI**	7.9±2.3	7.8±2.3	5.7±1.9	8.0±2.3	-	-	-	-	-	-	-	-	-	-	-	-	-
**TALA**	7.1±2.3	7.2±2.4	5.1±1.8	7.2±2.2	1.4±0.9	-	-	-	-	-	-	-	-	-	-	-	-
**TTON**	4.3±1.8	4.8 ±2.1	1.5 ±1.3	5.4 ±2.1	10.7±2.7	10.7±2. 9	-	-	-	-	-	-	-	-	-	-	-
**TOBE**	2.6±1.2	4.0±1.7	2.7 ±1.3	3.1 ±1.5	8.8 ±2.3	8.0 ±2.3	5.7±2.1	-	-	-	-	-	-	-	-	-	-
**KPEL**	24.3±3.7	23.6±3.6	19.6±3.1	24.1±3.6	24.1±3.5	24.2±3.6	24.8±3.6	21.9±3.6	-	-	-	-	-	-	-	-	
**EALL**	26.3±3.7	25.9±3.7	17.2±2.9	21.4±3.2	23.1±3.3	24.7±3.7	28.8±3.9	24.7±3.6	21.4±3.5	-	-	-	-	-	-	-	-
**ATHA**	28.7±3.8	28.1±3.7	20.7±3.0	25.8±3.5	23.3±3.4	25.1±3.8	31.8±4.0	27.4±3.8	21.7±3.6	19.5±3.4	-	-	-	-	-	-	-
**AROC**	26.6±3.8	25.5±3.8	17.8±2.8	22.9±3.2	21.6±3.1	25.6±3 .7	28.1±3.9	24.2±3.7	17.0±3.3	16.1±3.2	12.0±3.0	-	-	-	-	-	-
**SSAR**	23.6±3.8	24.1±3.8	18.9±3.2	21.8±3.5	17.7±3.2	17.7±3.4	27.7±4.0	22.3±3.6	16.7±3.4	22.9±3.5	21.3±3.5	22.4±3.8	-	-	-	-	-
**SSCO**	33.3±4.0	33.5±4.0	25.2±3.4	31.7±3.7	29.4±3.7	30.4±3.9	35.6±4.1	35.2±4.0	34.7±3.9	33.8±3.9	34.9±4.0	32.8±4.0	33.8±4.0	-	-	-	-
**SJAP**	31.5±4.1	29.2±3.9	24.4±3.5	27.8±3.8	28.3±3.8	30.3± 4.1	33.7±4.2	30.3±4.0	30.2±3.9	27.9±3.7	31.4±3.8	29 .5±3.9	29.6±4.0	27.1±3.8	-	-	-
**SCOL**	35.3±4.5	32.9±4.3	28.6±3.9	32.4±4.3	33.8±4.2	34.9±4.4	37.9±4.5	35.1±4.5	32.8±4.2	31.4±4.0	36.2±4.0	34.0±4.0	35.1±4.4	28.7±3.9	4.5±1.5	-	-
**SAUS**	26.8±3.6	25.4±3.4	20.8±3.0	23.7±3.2	25.5±3.3	26.7±3 .7	28.6±3.6	26.1±3.4	26.1±3.4	24.7±3.3	27.8±3.5	27. 0±3.5	26.1±3.6	22.7±3.3	1.7±1.1	2.8±1.1	-
